# “My First Threesome with Them Was a Religious Experience”: A Mixed-Methods Study of Symbiosexual Experiences

**DOI:** 10.1007/s10508-025-03095-5

**Published:** 2025-02-21

**Authors:** Sally W. Johnston

**Affiliations:** Human Sexuality Department, California Institute of Integral Studies, San Francisco, CA 94103 USA

**Keywords:** Symbiosexual, Polyamory, Non-monogamy, Sexual behaviors, Monosexuality

## Abstract

People of diverse backgrounds and identities report experiencing attraction to the energy, multidimensionality, and power created by people in relationships (Johnston, 2024a). Due to cultural privileging of monosexuality and monogamy, and stigma specifically within polyamory communities against people interested in sex and relationships with couples, very little is known about this population. Lack of recognition and validation negatively impacts those who experience this attraction, known as symbiosexual attraction (Johnston & Schoenfeld, 2021). I conducted a mixed-methods analysis of secondary data from The Pleasure Study (Harvey et al., 2023) to investigate the sexual and romantic experiences of this population. I found that people who experience symbiosexual attraction engage in a variety non-monogamous, multi-person sexual and relationship dynamics. Those who engage in these dynamics specifically with couples report a variety of unique and heightened experiences, both gratifying and undesirable. These findings fill a gap in the literature on this largely unexamined population and challenge the stigma in the polyamorous community as well as within our broader mononormative culture against sex and relationships with established couples which may be preventing people from engaging in ways that best align with their desires and center their pleasure.

## Introduction

If we understand ourselves as “erotic,” rather than (self-evidently, or universally) “sexual,” our creatureliness has a different valence. Possibility is at the heart of this conception of humanness. (Willey, 2016, pp. 128-129)

While in the last few decades we have recognized sexuality labels to address multi-gender preferences, like bisexuality and pansexuality, a vast ocean of multi-person erotic possibilities remain unnamed and unaccounted for in the lived experience of human sexuality in the Western world. One such desire that has yet to be fully explored in academic literature is the individual experience of attraction to people in relationships. A recent study found that some people report experiencing attraction not to individual people but to the energy, multidimensionality, and power created by people in relationships (Johnston, 2024a). This experience of attraction, known as symbiosexual attraction (Johnston & Schoenfeld, 2021), is traceable in both Western and non-Western discourses and is reported by people of diverse backgrounds and identities (Johnston, 2024a, 2024b). Further, people experience this desire strongly, frequently, and/or pervasively (Johnston, 2024a).

The nature of symbiosexual attraction, as an erotic experience that is not monosexual and not centered on gender, has rendered it largely invisible culturally and academically. Paradoxically, symbiosexual attractions receive heightened attention and are uniquely stigmatized within polyamorous communities. Women who express interest in sex and relationships with couples (sometimes known as unicorns) are stigmatized as emotionally unhealthy for having this desire and criticized for being uneducated about the unethical, abusive power dynamics polyamorous people believe are inherent in sex and relationships with couples (Johnston, 2024c). Due to cultural and academic invisibility, the multi-person sexual and romantic experiences of people who experience symbiosexual attraction is unknown. Further the assumed biases and critiques (often aimed at so-called unicorns) are left unproven.

Through a queer feminist lens, this study explores the prevalence and nature of multi-person sexual and romantic experiences of people who experience symbiosexual attraction. Considering the prevalence of multi-partner sexual and relationship preferences and behaviors including threesomes, group sex, and non-monogamous relationship dynamics such as triads and polycules (Herbenick, 2017; 2021; Lehmiller, 2018; Rubel & Burleigh, 2020; Scoats, 2020; Sheff, 2014) and evidence of symbiosexual attraction (Johnston, 2024a), lack of studies on the intersection of multi-person attractions and multi-person sexual and romantic experiences is surprising. Further, existing studies on multi-person sexual behaviors typically track for risk level instead of pleasure (Westbrook et al., 2022). Assumptions of multi-person desires and behaviors as risky aberrations not only ignore pleasure but the importance of these experiences to one’s sexual life.

Lack of recognition and validation negatively impacts those who experience symbiosexual desires. In her studies of plurisexualities, sexualities oriented toward multiple people like bisexuality and pansexuality, Hayfield (2021) found that those with plurisexual desires face ongoing marginalization and discrimination due to cultural assumptions and privileging of monosexuality and monogamy. These discriminations and marginalizations, which have been linked to decreased health and wellbeing and trouble with sense of self (Hayfield, 2021), extend not just to attractions to multiple genders but to all non-monosexual desires and experiences. Considering the harms associated with non-monosexual marginalization and discrimination, and the stigma of symbiosexual desires within the polyamorous community, research on the lived experiences of sexuality for those with symbiosexual attractions is warranted.

With very limited research on this population, I conducted an exploratory mixed-methods analysis (Bryman, 2016; Fetters et al., 2013) of secondary data from *The Pleasure Study* (Harvey et al., 2023) to examine sexual and romantic experiences of those who experience symbiosexual attraction. This study engages Westbrook et al.’s (2022) recommendation to consider and include qualitative measures of outcomes (e.g., experiences of pleasure and satisfaction as well as experiences dissatisfaction and harm) as a critical component of a person’s lived experience of sexuality. Studying the sexual and romantic experiences of people who experience symbiosexual attraction will fill a gap in knowledge on this largely unexamined population and test the validity of the stigma against sex and relationships with established couples—a stigma pervasive in the polyamorous community as well as within our broader mononormative culture. Studying these experiences may also challenge harmful assumptions that prevent people with symbiosexual attraction from engaging in sex and relationships that align with their desires and center their pleasure.

### Research Questions

I analyzed both qualitative and quantitative data from *The Pleasure Study* (Harvey et al., 2023) to address the following research questions about symbiosexual experiences:

- What are the sexual and relationship behaviors of people who experience symbiosexual attraction?
- Do they engage in non-monogamous, multi-partner sex and relationship dynamics? How prevalent are these experiences?
- Do those specifically attracted to couples engage in sexual and romantic experiences with couples? If so, how frequently?
- For those who engage in sex and relationship dynamics with couples what is the prevalence of gratifying and undesirable experiences? What kinds of experiences are described positively versus negatively?

## Method

With limited research on symbiosexuality, I conducted an exploratory mixed-methods analysis of secondary data from Stage 2 of *The Pleasure Study* (Harvey et al., 2023) to examine sexual and romantic experiences with couples for those who experience symbiosexual attraction. *The Pleasure Study* was designed to investigate the relationship between gender identity/expression and sexual pleasure. In Stage 2, researchers sought to investigate why femininity and those with marginalized gender identities are associated with increased performances of sexual pleasure, the primary finding from stage one (Harvey, 2020, 2021). Stage 2 included questions that inquired about gender, sexual orientation, relationship practices, culture, education, performance of sexual pleasure (i.e., performing/faking orgasm, performing/faking pleasure with sounds such as moaning or gestures such as back arching or muscle clenching) and specifically included questions about romantic and sexual experiences with couples.

### Participants

In Stage 2 of *The Pleasure Study* (Harvey et al., 2023), researchers specifically recruited queer (LGTBQ) and non-monogamous populations using convenience sampling and snowball sampling (Dunne, 2002). Participants were recruited from online community spaces (such as *Facebook*, *Reddit*, *Instagram*, *Meetup*, and community listservs) using digital flyers and posts advertising the study and including a link to the survey. Participants had to be English speaking and express their consent prior to participating in the survey. Participants were excluded from the study if they were under 21 or reported that they never had sex. (Sex was self-defined.)

I analyzed data that was collected up until May 2023 from Stage 2 of *The Pleasure Study*. The sample included a total of 373 survey participants and 42 interviewees. (Interviewees were part of the survey sample.) The sample included a larger than average portion of queer sexualities (74.4%) and genders (35.7%), as well as non-monogamous relationship identities (75.0%). Participants were predominantly between 21 and 40 years old (75.5%), White (66.6%), not religious (74.7%), attained a bachelor’s degree or higher (69.5%), middle class (50%), living in the USA (70.4%), and living in urban areas (83.8%).

For the current study, I included all survey participants from the sample described above who indicated on the survey previous attraction to a couple. The sample, a total of 145 survey participants (38.9% of *The Pleasure Study* survey participants), included a large percentage of queer sexualities (90.3%) and genders (34.5%), as well as non-monogamous relationship identities (87.5%). Participants were predominately between 21 and 40 years old (74.4%), White (66.4%), not religious (78.0%), attained a bachelor’s degree or higher (71.1%), middle class (47.4%), living in the USA (65.5%), and living in urban areas (83.7%). See Table 1 for the demographic distributions of the study sample.

**Table 1:** Survey respondents who experience symbiosexual attraction

Variable	Frequency	Valid percentage
Age (in years)		
21–30	43	33.3%
31–40	53	41.1%
41–50	20	15.5%
51–60	11	8.5%
61–70	2	1.6%
Missing	16	
Race/Ethnicity^a^		
Asian/South Asian	6	4.4%
Arab	0	0.0%
Black/African American	15	11.0%
Hispanic/Latinx/Spanish	12	8.8%
Indigenous Peoples^b^	8	4.8%
Jewish	10	7.3%
Middle Eastern/North African	0	0.0%
White (Only)	91	66.4%
Missing	8	
Religion		
Important	29	22.0%
Christian/Catholic	9	
Jewish	5	
Pagan/Witchcraft	10	
Not Important	103	78.0%
Missing	13	
Education		
Less than High School	0	0.0%
High School/GED	2	1.5%
Some College/Associates	37	27.4%
Bachelors	45	33.3%
Masters	41	30.4%
Doctoral/Professional	10	7.4%
Missing	10	
Social Class		
Working	48	36.1%
Middle	63	47.4%
Upper/Middle, Upper	22	16.5%
Missing	12	
Country/Region		
U.S. (29 States)	78	65.5%
Mid-West	19	
North-East	16	
South-East	20	
South-West	5	
West	18	
Outside U.S. (9 Countries)	41	34.5%
Australia	1	
Canada	30	
Germany	2	
India	1	
Ireland	1	
Luxembourg	1	
Peru	1	
Portugal	1	
UK	3	
Missing	26	
Community Type		
Rural	22	16.3%
Urban	113	83.7%
Missing	10	
Sexual Orientation^c^		
Asexual Only	1	0.7%
Heterosexual Only	14	9.7%
Gay, Gay/Queer Only	8	5.5%
Lesbian, Lesbian/Queer Only	4	2.8%
Bisexual, Bisexual/Queer Only	34	23.4%
Pansexual, Pansexual/Queer Only	26	20.0%
Other (Bi/Pan/Queer, etc.)	58	40.0%
Gender Identity^d^		
Man Only	33	22.8%
Woman Only	62	42.8%
Other (genderqueer, non-binary,trans)	50	34.5%
Relationship Identity		
Monogamous	18	12.5%
Non-monogamous	126	87.5%
Polyamorous	97	67.4%
Missing	1	

Data from Stage 2 of The Pleasure Study (Harvey et al., 2023), *N* = 145

^a^Respondents were able to choose multiple race/ethnicities

^b^On the survey the race/ethnicity options were labeled American Indian/Alaskan Native and Native Hawaiian/Pacific Islander

^c^Respondents were able to choose multiple orientations

^d^Respondents were able to choose multiple gender identities

I also included data from all interviewees who indicated on *The Pleasure Study* survey previous experience of attraction to a couple. The sample, a total of 34 interviewees (81.0% of *The Pleasure Study* interviewees), included diverse ages, race/ethnicities, religions, social classes, nationalities, regions, community types, sexualities, genders, relationship identities. See Table 2 for individual interviewee demographic information.

**Table 2:** Survey data of individual interviewees who experience symbiosexual attraction

Pseudonym	Demographic data					
	Gender	Sexuality	Relationship identity	Race	Age	Location
Amari	Non-binary	Queer/skoliosexual	Polyamorous	White	39	Georgia (urban)
Angel	Non-binary/ woman/dyke	Bisexual/lesbian/ queer	Polyamorous	White/Jewish	26	Illinois (urban)
Asa	Woman	Bisexual/pansexual	Polyamorous	Hispanic/White	32	Texas (urban)
Avery	Trans man	Queer	Monogamous	White	28	Michigan (urban)
Bellamy	Non-binary/ trans/femme	Bisexual/gay/ pansexual/queer	Polyamorous	Asian/White	27	Mississippi (rural)
Blake	Man	Gay	“Currently monogamous”	White	28	Michigan (urban)
Cameron	Man	Asexual/gay/ queer	“Currently nonmonogamous”	White	35	Ohio (urban)
Casey	Trans woman	Bisexual/pansexual /queer	“Preferably monogamous”	White	missing	Iowa (urban)
Charlie	Woman	Pansexual	Polyamorous	White	42	California (rural)
Devin	Man	Heterosexual/ heteroflexible	Non-monogamous	White	32	California (urban)
Drew	Man	Heterosexual	Polyamorous	White	45	Illinois (urban)
Eden	Man/ “want to be genderfluid”	Bisexual/pansexual /queer	Polyamorous	White	30	Illinois (urban)
Ellis	Non-binary/ woman	Bisexual/pansexual /queer	Polyamorous	White	33	Massachusetts (urban)
Harlow	Non-binary/ trans	Queer	Polyamorous	Jewish/White	34	New York (urban)
Hayden	Woman	Queer	Ethically non-monogamous	White	52	Canada (urban)
Kamari	Man/bigender	Queer/polysexual	Polyamorous	Black (Caribbean)	50	Canada (urban)
Kendall	Non-binary/ woman	Bisexual	Monogamous	White	27	Indiana (rural)
Lennon	Non-binary/ genderqueer	Queer	Ethically non-monogamous	White	30	New York (urban)
Logan	Woman	Bisexual/queer	Monogamous	Hispanic	23	Florida (rural)
Noa	Non-binary	Queer	Polyamorous	White/Jewish	25	Oregon (urban)
Onyx	Non-binary	Bisexual	Polyamorous	White	39	UK (rural)
Parker	Non-binary	Pansexual/queer	Ethically non-monogamous	White	36	California (urban)
Phoenix	Woman	Bisexual	“Situationally” monogamous/ Polyamorous	White	23	Germany (urban)
Peyton	Woman	Bisexual	Ethically non-monogamous	Asian/White	30	California (urban)
Quinn	Non-binary	Lesbian	Polyamorous	White	23	Kentucky (urban)
Reece	Woman	Bisexual	Polyamorous	White	48	Canada (urban)
Riley	Woman	Pansexual	Polyamorous	White	55	Canada (urban)
River	Non-binary/ trans man	Pansexual/queer	Polyamorous	White	34	Virginia (urban)
Rowan	Man	Pansexual	Consensually non-monogamous	White	59	Minnesota (urban)
Sage	Non-binary	Pansexual	Polyamorous	White	39	Missouri (urban)
Sawyer	Woman	Bisexual/queer	Ethically non-monogamous	White	35	Luxembourg (urban)
Skyler	Woman	Heterosexual	Ethically non-monogamous	Asian	40	Canada (urban)
Taylor	Trans woman	Sapphic bisexual	Polyamorous	White	missing	Oklahoma (urban)
Teagan	Man/non-binary/ transman/woman/ Nonconforming	Pansexual/queer	Polyamorous	Indigenous Peoples^a^/White/“Culturally multicultural, mostly black”	49	Illinois (rural)

Data from Stage 2 of the Pleasure Study (Harvey et al., 2023), *N* = 34

^a^On the survey, the race/ethnicity options were labeled American Indian/Alaskan Native and Native Hawaiian/Pacific Islander

### Measures and Procedure

*The Pleasure Study* survey instrument consisted of 65 questions and took participants approximately 20 min to complete online. The survey included a mix of multiple choice, Likert scale, and open-ended questions about gender, sexual orientation, relationship practices, culture, education, performance of sexual pleasure and specifically included questions about experiences with couples. The study also included interviews with those who indicated (in the survey) willingness to participate in an interview. Researchers conducted interviews over Zoom, lasting between 1 and 2 h. Participants answered semi-structured questions about their gender, orientation, relationship practices, culture, education, and performance of sexual pleasure, as well as questions about romantic and sexual experiences with couples. Audio files recorded via Zoom were transcribed using Otter AI Pro transcription software. Transcripts were cleaned and anonymized by myself (as the research coordinator for *The Pleasure Study*) and *The Pleasure Study* research assistants.

### Data Analysis

I analyzed both survey and interview data from *The Pleasure Study* for this mixed-methods study (Bryman, 2016). By using a mixed-methods design, I accessed the strengths of each method to investigate an under studied and inherently complex phenomenon like symbiosexuality. For research question one, I analyzed frequency tables derived from quantitative data using SPSS Version 28. For research question two, I analyzed qualitative data (from the interviews) for thematic content. Using thematic analysis (Bryman, 2016), I coded and quantified qualitative data from the interviews relevant to the nature of sexual and romantic experience with couples as gratifying or undesirable. Once coded (as gratifying and/or undesirable), I analyzed gratifying and undesirable qualitative data for thematic content (Bryman, 2016). I organized gratifying and undesirable themes based on interviewee reports of their experiences with couples.

For the purpose of this study, I operationalized symbiosexual attraction as the experience of attraction to a couple. All participants included in the study answered yes on the survey to the question: Have you ever felt sexually/romantically attracted to a couple (two people and their relationship together, not each of them individually)? Survey questions about the prevalence of non-monogamous and multi-partner sexual and romantic experience, the frequency of sexual and romantic experiences with couples (Likert-type scale never to often), and demographic questions (gender, sexual orientation, and relationship identity were included in analysis (see Appendix A). Interview questions about sexual and romantic experiences with couples were also included in analysis (See Appendix B).

## Results

### Research Question 1: What Are the Sexual and Relationship Behaviors of People Who Experience Symbiosexual Attraction?

A total of 145 (38.9%) of the 373 Pleasure Study survey respondents reported experiencing symbiosexual attraction, specifically attraction to couples. Of the 145 respondents, 130 (89.7%) reported engaging in a variety of non-monogamous, multiparter relationship dynamics. The high percentage of these experiences and choices may directly relate to symbiosexuality as an experience that is recognized through non-monogamous, multi-partner dynamics and/or as an attraction that motivates non-monogamous, multi-partner sex and relationship choices. Of the 143 respondents who answered the questions specifically about sexual and romantic experiences with couples, 101 (70.6%) respondents reported engaging in sexual experiences with couples, and 85 (59.4%) respondents reported having had romantic/dating experiences with couples. While these percentages are high, for a population that specifically experiences attraction to couples they highlight a notable gap between desire and behavior.

### Do They Engage in Non-Monogamous, Multi-Partner Sex and Relationship Dynamics? How Prevalent Are These Experiences?

**Fig. 1:**
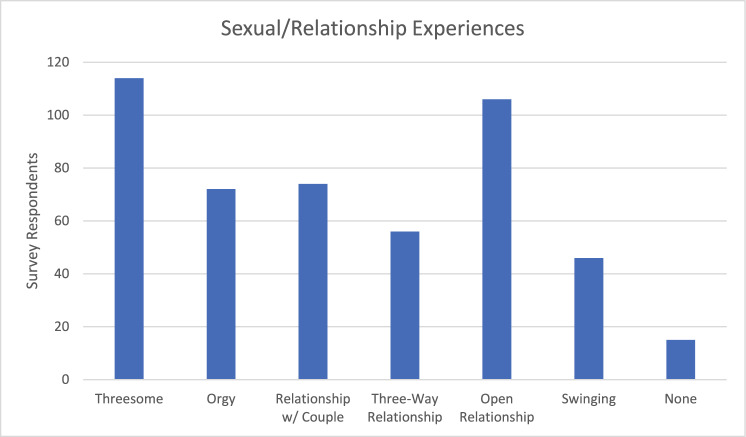
Sexual and relationship experiences of people who experience symbiosexual attraction note. Data from Stage 2 of the pleasure study (Harvey et al., 2023). N = 145

*Prevalence of Non-Monogamous Sex and Relationship Experiences*. Survey respondents reported a variety of experiences with non-monogamous or multi-partner sex and relationship dynamics. A total of 114 respondents (78.6%) report having had a threesome, 72 (49.7%) report having had an orgy (sex with more the two people), 74 (51.0%) report having had a relationship with a couple, 56 (38.6%) report having had a three-way relationship, 106 (73.1%) report having had an open relationship, 46 (31.7%) report having had swinging experiences, and 15 (10.3%) report that they have had none of these experiences (see Fig. 1). The prevalence of these different experiences aligns with both the social acceptability of each dynamic and the ease with which each dynamic may be realized (e.g., threesomes are more socially acceptable and easier to configure than orgies and open relationships require less group consensus than a three-way relationship) (Beggan, 2020).

### Do Those Specifically Attracted to Couples Engage in Sexual and Romantic Experiences with Couples? If So, How Frequently?

**Fig. 2:**
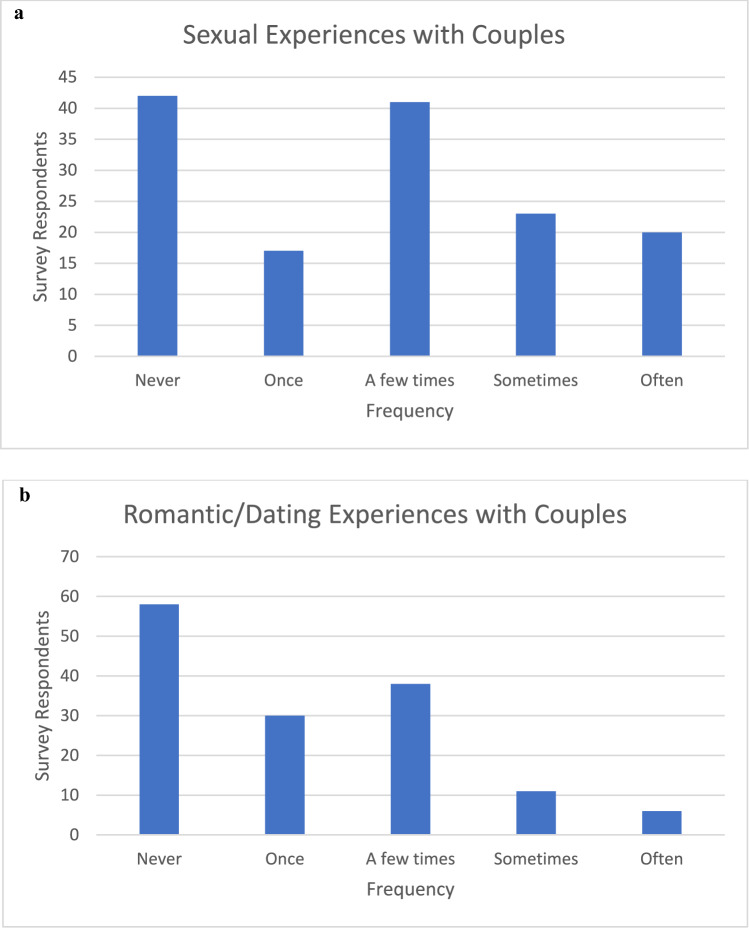
**a**: Frequency of sexual experiences with couples for people who experience symbiosexual attraction Note. Data from Stage 2 of The Pleasure Study (Harvey et al., 2023). N = 143. **b**: Frequency of romantic/dating experiences with couples for people who experience symbiosexual attraction Note. Data from Stage 2 of The Pleasure Study (Harvey et al., 2023). N = 143

*Frequency of Sexual Experiences with Couples*. Forty-three survey respondents (30.1%) reported having sexual experiences with couples sometimes or often, indicating that, for some people who experience symbiosexual attraction, sexual dynamics with couples is a significant part of their lived experience. Forty-one survey respondents (28.7%) reported having sexual experiences with couples a few times. Seventeen survey respondents (11.9%) reported having sexual experiences with couples once. Forty-two survey respondents (29.4%) reported never having sexual experiences with couples, indicating that, for some people with symbiosexual attraction, a larger force (a more primary sexual desire or the social acceptability of that desire) or limited availability is motivating their sexual choices (see Fig. 2a).

*Frequency of Romantic/Dating Experiences with Couples*. Seventeen survey respondents (11.9%) reported having romantic/dating experiences with couples sometimes or often, indicating that, for some people who experience symbiosexual attraction, relationship dynamics with couples is a significant part of their lived experience. Thirty-eight survey respondents (26.6%) reported having romantic/dating experiences with couples a few times. Thirty survey respondents (21.0%) reported having romantic/dating experiences with couples once. Fifty-eight survey respondents (40.6%) reported never having romantic/dating experiences with couples indicating that again for some people with symbiosexual attraction a larger force (a more primary romantic desire or the social acceptability of that desire) is motivating their relationship choices (see Fig. 2b).

### Research Question 2: For Those Who Engage in Sex and Relationship Dynamics with Couples What Is the Prevalence of Gratifying and Undesirable Experiences? What Kinds of Experiences Are Described Positively versus Negatively?

Thirty-four Pleasure Study interviewees reported experiencing symbiosexual attraction. Of the 34, 27 (79.4%) reported having sexual and/or romantic experiences with couples. Twenty (74.1%) described gratifying experiences. Nine (33.3%) described undesirable experiences. There was an overlap of percentages because six (22.2%) interviewees described both gratifying and undesirable experiences. These findings suggest that, just like monosexual, monogamous sex and relationship dynamics, a range of experiences are possible in dynamics with couples. Further, the number of gratifying experiences described by interviewees challenges the stigma within polyamory communities that sexual and romantic dynamics with couples are inherently negative and harmful.

### Gratifying Experiences

I found rich detail in interviewee descriptions of their sex and relationships with couples about the gratifying experiences made possible through these dynamics. Gratifying experiences included heightened pleasure and fun, the opportunity for playing with roles, gender expansiveness, the opportunity to be objectified or have the “unicorn” experience, heightened intimacy and closeness, and the opportunity for healing/transformation. These expansive, pleasurable experiences with couples unfolded in very enticing and sometimes mind-blowing ways. For some interviewees, the pleasure and possibility offered through sexual and romantic dynamics with couples, symbiosexual dynamics, was unmatched and sparked a self-actualizing experience of their sexuality and personhood. As Kamari (50, queer/polysexual, polyam, man/bigender) explained, they found that, unlike sex and relationships with individuals, the multiple perspectives offered through sex and relationships with couples allowed couples to “fully see me for the person who I am.” Kamari felt recognized for their full humanity because each member of the couple brought different perspectives to sexual and romantic encounters. Kamari’s couple shared their perspectives with one another multiplying the sexual and relational information with which they could use to more fully engage with Kamari. In turn, this multiplied Kamari’s level of engagement with the couple.

While symbiosexual dynamics provided gratifying benefits of complexity, multidimensionality, and heightened energy and emotion, these dynamics also proved challenging to navigate both within the inter-relationships of three people and within our broader Western culture that greatly values monosexuality and monogamy. As Lennon (30, queer, ENM, non-binary/genderqueer) explained, because they tend to engage in sex and relationships with “older, established married couples” they felt they were often working against a “pattern.” They described this pattern as one where couples were quick to retreat together from triadic dynamics and dismiss the third person. They attributed this pattern to the amount of “undoing” that engaging in these dynamics required of couples in “our very monogamous, normative society.” While this interviewee greatly valued their symbiosexual experiences, they understood and accepted that such dynamics come with inherent, understandable and, in some cases insurmountable, structural challenges.

*Heightened Pleasure/Fun*. Many interviewees talked about how engaging sexually with couples increased their experiences of pleasure. Entering the sexual space of two people with their own sexual history heightened the sexual energy, multiplied the sensory experiences, and ramped up the excitement and fun of the interaction. Further, the dynamics of couples offered more possibilities for spontaneity, fluidity, and play. As Reece (48, bisexual, polyam, woman) explained about sex with couples:

Gives you a chance to try things that you can't do just with two people. Two people can be pleasuring you at once, you can have multiple things being pleasured at the same time. You've never had multiple people to be pleasured at the same time, which wakes up all different feelings within yourself and for them too…It’s a different energy when it's with a couple…to be able to be in tandem with people like that…was really hot and really fun because again, it's totally different.

Also speaking to the heightened sensory experiences of being with two people, Hayden (52, queer, ENM, woman) explained how “there's more of an audible or visual stimuli” when you play with couples. Another interviewee Charlie (42, pansexual, polyam, woman) talked about the heightened stimuli with multiples bodies and how hot sex with a particular couple was:

It's so hot. I mean, honestly, the sex that the three of us have together is always incredible. It is just, we're all just exhausted afterwards…If I'm going to go to a spank bank moment in my head in the shower or something, it's them, which is so much fun…I love also watching my partners receive pleasure and watching my partners give pleasure and the only way I could do that is to have a third person…It's really fun to just get to experience all of the parts. Pussies are amazing, penises are great, ass holes are fantastic. To get all of them and breasts and mouths and you know... it's different when you're having sex with two people who are also already connected, and they know themselves really well. And then you can learn about them and their partners so much faster because you've got the inside track there, you've got someone telling you “well they like this and they like that” and “let me show you how I make them squirt.”

Charlie attributed their heightened pleasure not only to the added stimuli of symbiosexual dynamics but to the body knowledge people in relationships have of one another and how that knowledge can greatly enhance sexual experiences.

The pleasure of sex with couples was perhaps best summed up by Peyton (30, bisexual, ENM [Ethically non-monogamous], woman) who explained of her sexual experiences with one specific couple, “It was just overwhelming pleasure. The most pleasure I've had, ever felt in my life up until that point where I was just like I cannot believe that life can be this good.” Peyton highlighted what several interviewees experienced, sexual self-actualization through their experiences of pleasure in symbiosexual dynamics. This truer, more gratifying experience and expression of sexuality allowed her to see life itself differently, better.

*Fluid Role Play*. Interviewees also specifically highlighted role play as a gratifying experience both in the diversity and fluidity of roles offered through symbiosexual dynamics, as well in the multiplied power of dominant and submissive experiences created through the aligning or polarizing expressions of power in the members of the couple. Reece (48, bisexual, polyam, woman) described that they loved that in sex and relationships with couples there are no set roles: “I found it's really exciting not necessarily knowing how it's going to function.” Also speaking to role fluidity and responsiveness in symbiosexual dynamics, Hayden (52, queer, ENM, woman) explained how the focus shifts with couples:

If it's one on one, you're only focused on each other. When you're with multiple people, your focus changes throughout the session. And I think you're thinking more about equality, or equity, when you are with multiple people to make sure everybody's getting equal time, equal pleasure…. Everybody's got different needs and different things that they like so you kind of have to change it up. You have to change it up, depending on who's participating. I think there's a lot more room for spontaneity.

The appeal of variety and diversity offered through role play was also shared by Eden (30, bisexual/pansexual/queer, polyam, man) who wants “to be genderfluid” but unsure how to do so “respectfully and safely.” He explained that his “ideal life” would be sex and “short term” relationships with couples because, “I can serve all these different roles. I can engage in different acts with them. It just it seems all like a net positive.” Charlie (42, pansexual, polyam, woman) elaborated on how this variety can include a shift of power. She talked about this specifically with one couple with whom she has sex. Charlie explained that she loved how “we do a lot of role-play together…we experiment a lot with each other, which is really fun. We can take turns dominating and being more bottom.” With an extra person, two-person sexual scripts (Gagnon & Simon, 1973) fall away in favor of something more improvisational.

A few interviewees specifically talked about enhanced Dom/Sub role play possibilities in symbiosexual dynamics. Cameron (35, asexual/gay/queer, “currently non-monogamous,” man) described a “good experience” where he got to playout his preferred role as a Dom, with “two men who were in a relationship together and who were both more submissive and wanted basically just experimentation of both being submissive to someone else in kind of a kink role-play.” Similarly, Ellis (33, bisexual/pansexual/queer, polyam, non-binary/woman) described a specific experience with a couple where she had “a great time” playing a submissive role along with one member of the couple and seeing how the more dominant partner reacted to multiple submissive energies. These interviewees described experiences that offered stronger and more varied power dynamics in the dominant or submissive roles which appealed to them.

*Gender Expansiveness.* Expanding on the potential of role play, interviewees also talked about how they greatly valued the opportunity to play with, expand, or undo gender roles in symbiosexual dynamics. Kamari (50, queer/polysexual, polyam, man/bigender) explained how “delicious” and bigender-validating he finds the flow of masculine and feminine energies together in sex with different gendered couples:

I would say that all my experiences with couples have been quite positive... I find that the couples that are open to me…There's just been an opening of you know a flow of energy between the three of us…The male will get the female perspective. The female gets the male perspective. So, they get a full perspective of you. Where, if you have either one, and they're only looking at it through their one perspective, so that's the difference.

While Kamari enjoyed more wholeness of their gender expression in symbiosexual dynamics, Rowan (59, pansexual, CNM, man) enjoyed how these experiences operated to undo gender. Rowan explained how he loved how gender became irrelevant in his sexual and romantic experiences with couples and how he loves the “gender fuckery” and “gender switch” opportunities of these experiences. He spoke specifically about the opportunity for both experience and expression of vulnerability available for men in symbiosexual dynamics, particularly for men who feel and demonstrate “compersion” for their female partners.^1^

Describing a similar expanded or softened experience of masculinity, Blake (28, gay, “currently monogamous,” man) explained of the relationship that he “tried” with a man and a woman, he enjoyed that he, as a third to a couple, had “an outsider’s view” to observe how this man treated this woman and:

The way the man ditched scripts of toxic masculinity.... There was always that aspect of the guy who really respected the girl and understood what she wanted and did his best to support her and was not toxically masculine…which was different, as far as my interactions with other men in high school.

Just as pleasure and satisfaction were heightened through power subversion in role play, gender play specifically offered potential for pleasure and satisfaction in the subversion of power in gendered sex and relationships.

Expansiveness of the gendered experience also applied to specific dynamic preferences. Some interviewees identified specific gender combinations that most appealed to them in couple dynamics or best provided the container they wanted to playout their own expressions or expansions of gender. Charlie (42, pansexual, polyam, woman) explained that dynamics with a couple are “especially” great when the couple has different gender identities, “like when you have a man and a woman. I've also had interactions with a cis man and a trans woman and gosh that was so much fun.” Similarly, Angel (26, bisexual/lesbian/queer, polyam, non-binary/woman/dyke) described one relationship with a couple where they felt “really seen” and “really comfortable” being their genderqueer and sexually queer self because the couple they dated were “two non-binary people. Both of whom presented as butch or masc of center” and that made space for them to be themselves. Angel goes on to say the “sex was really fun. I had a really nice time.” These interviewees seem to be expressing specific multi-object or more accurately multi-gendered attraction: an orientation to specific mixes or expanded gender expressions or presentations offered through symbiosexual dynamics that increased sexual satisfaction and pleasure.

**Objectification/Unicorn Experience.** Some interviewees specifically spoke about how the power of the couple in symbiosexual dynamics created an erotic experience of objectification. Interviewees relished being immersed in a couple’s sexual world as the object of desire, fetish, fantasy, or “unicorn.” Lennon (30, queer, ENM, non-binary/genderqueer) explained that they enjoy being a unicorn and use that term “in an empowering sense.” They go on to explain that they enjoy when, “I am being used as a sexual object, and not in a way of like, I'm being objectified, I'm choosing to be this sexual object…I am that kind of extra flavor that's there at the moment.” Expressing similar delight in the heightened objectification offered through sex with couples, Charlie (42, pansexual, polyam, woman) explained:

I was what you call a unicorn in you know our modern language. And it was so much fun, you get to step into somebody else's fantasy and be that fantasy for them. And so, you are their pleasure center between the two of them and help them connect better.

She goes on to say, “they'll call me their sex doll which I fuckin love.” These interviewees makes a critical distinction between being objectified and choosing an experience of objectification in symbiosexual dynamics.

Interviewees who enjoyed objectification also enjoyed distance from the intensity of a subjective, emotional experience required between those committed to the romantic relationship. Devin (32, heterosexual/heteroflexible, NM, man) described how hot it was to be only the “vice” for pleasure sharing between a couple. Harlow (34, queer, polyam, non-binary/trans) referred to this position as “the shiny new object” and how they enjoyed the freedom to “dip in and out of a pre-existing relationship.” These interviewees seem to relish the role of the sexual object and of how symbiosexual dynamics both enhance that experience and add dimension to it. Through sex with couples, they could be both be more immersed in the position of the object than possible in one-to-one dynamics as well as more removed from the subjectivity and interdependence of the relationship of the couple, a space that may require emotion labor and, thus, can distract or decrease sexual desire and pleasure.

**Intimacy/Closeness.** Conversely, some interviewees talked about how much they appreciated the experience of heightened intimacy and closeness offered through symbiosexual dynamics. Sawyer (35, bisexual/queer, ENM, woman) explained about one couple she dated:

I really liked the way they interacted. …There was nothing about it that made me feel I wasn't being appreciated. But I could see them, the way they would look at each other to kind of communicate whether something was alright or not, checking in with each other. And I liked that because I thought it shows that they care about each other, it shows that they connect with each other on this level, and they check in to see if people are okay... I had the sensation of being invited inside the intimacy that they shared, and it felt like a really privileged position to be in, it was really nice…I remember just sitting back and enjoying watching them because I really enjoyed this feeling that had been fostered of we're all here together, we're all so lucky to be here together, we're all really happy to be here together. I think that's endured. I mean, that's why I keep seeing them after three years because when I play with them, I really like the dynamic they have, and I feel really lucky that I get to be part of it…It's nice when you find that, when you find that kind of love between people, and then they invite you to share some of it.

Sharing this gratitude for intimacy offered though dynamics with couples, Devin (32, heterosexual/heteroflexible, NM, man) talked about one couple that he had a sexual relationship with:

There's a much deeper closeness now between us in our friendships, and that's something I really admire and I truly value. I feel like I've gained two really strong friends recently from those experiences that I don't know if otherwise I would have been able to have that level of friendship. I've just considered myself very lucky to be able to call them my friend.

The physicality of heightened intimacy with couples was also talked about. Kamari (50, queer/polysexual, polyam, man/bigender) explained:

Sleeping three to a bed is just fantastic. It's just, whoever is in the middle, and it can change during the night, it's just that closeness, that intimacy, it's just, that's quite huge.

For these interviewees, a stronger emotional and physical intimacy was made possible through their sex and relationships with two people who already shared intimacy and emotional connection. They spoke of a reverence for this connection, both as something that they sought to nurture and felt nurtured by.

*Healing/Transformative*. For some interviewees, the nurturing power of symbiosexual dynamics went to another level. Perhaps most intriguing in interviewee descriptions was their experiences of healing and personal transformation made possible through sex and relationships with couples. The couple created a unique container of love, intimacy, healing, and care for them. Interviewees expressed tenderness and gratitude for strong and immersive experiences of intimacy when they were invited into a healthy, loving relationship with a couple. Some of these experiences were so gratifying they facilitated healing from exposure to less healthy relationship dynamics as well as personal transformation and growth.

Parker (36, pansexual/queer, ENM, non-binary) talked directly about how they believe there is something therapeutic about being a close witness to a good relationship through symbiosexual dynamics. They talked specifically about one couple who has “one of the most beautiful relationships [they] know” and with whom they are planning a future and share strong mutual love. Parker explained that being around a healthy relationship like this can be “really healing.” Particularly because the more they have dated, “the more [they] appreciate how much of a fucking dystopian wasteland it is and how few people actually know how to have healthy relationships.” They recount how they spoke to their therapist about how if it is possible to have “secondhand trauma” from witnessing unhealthy dynamics, “then maybe you can also be healed by seeing something healing happen.” They go on to explain “it is incredibly healing, to be able to be like, oh, I'm rediscovering a belief in relationships, it really can be good, it can be healthy, people really can do these things.” Of the admiration, love, and long-term dynamic they have with this couple, they describe it as a “transcendent thing” that “brings joy to [their] life.”

Also speaking to this opportunity for healing and building healthy relationships through symbiosexual dynamics, Rowan (59, pansexual, CNM, man) talked about growing up in a family that did not communicate and how they were able to develop their communication skills through their relationship with a married couple, “the communication is amazing. That is something that is really, really great, because in some ways [dynamics with couples] forces you to do that.”

Speaking most directly to the transcendent, healing potential of being with a couple, Peyton (30, bisexual, ENM, woman) described her first experience:

We hooked up a lot over the course of a couple years. They were just, I don't know, I feel like my first threesome with them was a religious experience. It was like, I think about it as a transformative experience because it was incredible…there was just this in sync-ness between them. Where it was, I just felt like I was in good hands. I felt extremely safe…I felt really taken care of by both of them.

Peyton and several others were able to find both solace in the container of the couple through which they could heal relationship pain as well as transcend from less healthy relationship experiences about what is possible in love and intimacy. New relationship possibilities were inspired specifically and uniquely through experiences with people already in nurturing, caring relationships. Unlike one-to-one dynamics interviewees were able to get a preview of the nature of their potential relationship with a couple and assess relationship health and skill sets to determine if entering the relationship might be safe and nourishing for them.

*Challenges*. While still framed as part of an overall gratifying experience, those who described their experiences with sex and relationships with couples positively also described the unique or heighted challenges they faced in these dynamics, including how complicated the dynamics can be specifically with communication issues and challenges of attention and balance, as well as the challenge of working within the relational and sexual scripts (Gagnon & Simon, 1973) of a mononormative society.

Speaking to complication of couple dynamics in sexual experiences, Drew (45, heterosexual, polyam, man) talked about the struggle to balance attention. He explained that while he really enjoys the experience of sex with couples, with one specific couple:

I felt like I was just pulling double duty and doing a lot of work. And both of them wanted all of the attention from everyone to themselves... It's just so hard to balance. So yeah, so I think like it's hard to meet everyone's needs 100% of the time as much as everyone wants it... I feel like there's just a lot more things to juggle…It was just very, very, it's like way more challenging.

Harlow (34, queer, polyam, non-binary/trans) offered an explanation for the heightened challenges of symbiosexual dynamics, “anytime you have two people, there's actually three relationships because there's you, the other person, and then the relationship so then it gets very complicated the more people that you add.”

In addition to the internal challenges of sex and relationship with couples, interviewees addressed the sociocultural challenges of symbiosexual dynamics. Mononormativity loomed in interviewee descriptions of challenges and inevitably colored their sexual and romantic experiences. Lennon (30, queer, ENM, non-binary/genderqueer) who had extensive sexual experiences with couples talked about how while their experiences with couples were “positive for the most part” what happened in their experiences was:

They'll be great, we'll have a great…what I think is a great time, and they are expressing is a great time, we'll text a little bit afterwards, and they'll completely fall off the radar. Oftentimes I am a first experience. I tend to be that for people and that's fine with me, you don't owe me anything…I get it, I get you're undoing a lot, you're doing a lot of work…I feel like it is very, very hard within our very monogamous, normative society.

These interviewee descriptions spoke both to what must be navigated within symbiosexual dynamics, the unique challenges of triadic relationships with an established couple, as well as how that navigation is directed or made more difficult by the social conditions of monosexual and monogamous privilege.

### Undesirable Experiences

I found rich detail in the interviewee descriptions of their sex and relationships with couples about the undesirable experiences possible in symbiosexual dynamics. For interviewees who described undesirable experiences, similar challenges were mentioned to those in the gratifying descriptions, but these interviewees experienced less benefits or more harm from these experiences. Despite their interest in couples, hard feelings and emotional discomfort outweighed experiences of pleasure and intimacy. Further, some interviewees felt victimized and disrespected by couples who privileged their own connection over the connection they shared with them, affirming that the power cultivated in the established relationship of a couple can be greater than that of individuals and can potentially inflict greater harm.

*Hard to Navigate*. Some interviewees who engaged in symbiosexual dynamics ultimately felt that these dynamics were too hard to navigate. Phoenix (23, bisexual, “situationally monogamous”/polyam, woman) explained that despite her attraction to couples when it came to actually engaging in sex and relationships:

It's quite hard to actually do that. And the reality of not all people are open to something polyamorous or an open relationship. Yeah, it came with a lot of struggles. When I actually tried to put that into reality or when I tried to come to terms with the way I felt.

She elaborates on a specific experience with a male/female couple:

I felt a lot of attraction towards both of them…He suggested having a threesome at some point as more of a joke and we also tried to actually put that into action. But then I think that she found it uncomfortable at some point that she thought that he and I are quite close. And that made it um quite hard because you obviously can't overstep her boundaries. I also don't want to do that. And it's still a little hard navigating that sort of situation because you obviously also don't want to break the relationship or those sorts of things. But on the other hand, especially with him, we are quite close. And yeah, that makes it hard.

While Phoenix spoke to the difficulties when connections, feelings, and even language for certain dynamics do not align between members of the couple, Teagan (49, pansexual/queer, polyam, man/non-binary/transman/woman/non-conforming) spoke to the difficulty of navigating the feelings shared between couples. Teagan explained:

I met these people and…it was this inclusive thing, where it seems like any interaction with one of them was an interaction with both of them…But then the wife ended up getting like too insecure about stuff. And the husband answered a question wrong, according to her, and so she was insecure that he might leave her. And so basically, before anything even started because we had massive negotiation…It was a complex situation.

These interviewees highlighted the challenge of having to tolerate a greater importance placed on the feelings shared between the members of a couple than of themselves.

Another interviewee spoke to the extra energy required even if one is willing to accept inequitable considerations. Onyx (39, bisexual, polyam, non-binary) explained that despite having good experiences with couples:

Dating couples, specifically, it's something now knowing what I know, with more words and language, it will traditionally be quite stressful and I'm lazy. And that's quite a lot of navigation. I wouldn't say if it happened organically I'm not saying I would say no, but there's plenty of fish in the sea and plenty of ways to have relationships, all that can be meaningful and valid, and I’d probably sort of take a couple of steps back and go “do you know what? That’s a lot.” I don’t know if I’ve got time for all of the conversations I would have with most of the people I connect with, and then times it by two and then times that by “how are we doing this as a three?” And how we all navigate. Yeah. No.

For these interviewees, the complexity of emotions and energy required of symbiosexual dynamics which included extra protections and considerations for the built relationship of the couple felt painful or too difficult to engage with. Such navigations did not feel worth the investment.

*Upset/Uncomfortable*. Some interviewees who described undesirable experiences talked about greater feelings of discomfort and unease possible in symbiosexual dynamics. Reece (48, bisexual, polyam, woman) explained that while she has loved sexual experiences with couples, “I’ve been in a situation where one person is not really comfortable and you’re kind of doing it because that other person really wants you to and that’s not comfortable.” Reece mentioned that although she had good experiences with “a man and a woman couple and me” the one time she had sex with a male/male couple, “that situation was uncomfortable for me because of my own personal hang ups.” Reece’s experience suggests that discomfort may not only apply to what is happening in the dynamics within the couple and the third person but how what is happening in symbiosexual dynamics aligns with or challenges accepted gender roles within the dynamics of group sex.

One interviewee elaborated on how the feelings of discomfort and upset feelings reported by interviewees may arise in dynamics with couples. Amari (39, queer/skoliosexual, polyam, non-binary) explained that while they “love group sex,” in dynamics with couples:

Almost every time a unicorn happens, you end up more attracted to one partner, and then it doesn’t work out with the other and hard feelings happen. And eventually it settles out or it doesn’t. In one case, it settled out and another it didn’t. The other partner got super butthurt and resentful and all that…If the attractions just happened, then yeah, I’d be game. But I don’t intend to go out only with the couple all the time, ever again.

While Reece and Amari addressed the discomfort of individual people’s feelings within symbiosexual dynamics, Rowan (59, pansexual, CNM, man) talked about the discomfort of the combination of big feelings that happen specifically between members of a couple. He explained “there were times when I was with a married couple, where I got a little uncomfortable with some big emotion that just happens in marriages, sometimes.” His experience speaks to the powerful emotions and related discomfort that may correlate with the level of commitment and enmeshment between the established couple in symbiosexual dynamics. For these interviewees, the attachment built between members of a couple, and the couple’s commitment to maintaining it, seemed to be more problematic than additive to the sex and relationship dynamics.

*Hierarchy/Using the Third Person*. The most talked about undesirable experience in dynamics with couples was the experience of feeling used or subjected to couple’s privilege without consent. Harlow (34, queer, polyam, non-binary/trans) explained how, while they were currently in a healthy, loving relationship with a couple:

A lot of the times what happens is that couples think that they wanted to invite a third person into their relationship and then one or both of them decide that they don’t and so then you’re caught in some sort of fucked up fight…I think that there can be like a lot of hierarchy that can be extremely hurtful. It takes a certain kind of person to really understand the power dynamics in a couple plus a third relationship and how to support a third person and feeling included and understanding how included they actually want to feel. A lot of times, people can start using the third, both people, as their personal therapist or sounding board.

Adding to this feeling of being used, Teagan (49, pansexual/queer, polyam, non-binary/transman/woman/non-conforming) described an experience with a couple:

The wife wanted to basically utilize me as a recreational toy and like “no, once a month, we’re gonna go far away and stay at a hotel, and then we can play, but we’re still gonna be friends and our kids are all gonna still interact, and you’re still gonna come over. But nobody will know that we’re doing this, even our families.” And to me, I was like, if I’m going to be doing stuff with you guys, we’re making hot cocoa together, we’re all together, the kids are going to know that I’m kissing you on your forehead. If I’m here, I’m here. I’m not your fucking objectified little toy, because of your fantasy of whatever.

Teagan goes on to mention that they came to believe that the couple’s unity was actually “some trauma replaying over and over again, that I just decided I didn’t want to take part in and so I stepped away.”

Another interviewee also used the word toy to describe their experiences with couples. Ellis (33, bisexual/pansexual/queer, polyam, non-binary/woman) explained that while they are very interested in relationships with couples, they “unfortunately” have limited experience because they explain:

I find that most of the time when you find couples who are looking to date somebody, effectively it’s like a couple that wants a third person to be their toy, basically, not to be a full relationship partner with them or whatever. So that kind of puts a damper on actually dating, but I’ve definitely had sexual relationships with couples.

Even in the sexual dynamics Ellis explains:

I have to at least like the person and feel respected by the person. So, if I feel like I could just as easily be a sex toy, why am I even here? This should be about everybody involved having fun, not just the couple having fun by using me as a thing, just spice it up in the bedroom for them or whatever.

Angel (26, bisexual/lesbian/queer, polyam, non-binary/woman/dyke) explained how this feeling of being treated as less than, which interviewees associated with certain couples, was a product of the power dynamics. They explained that while they loved sex and relationships with couples, these experiences.

Haven’t ended well for me historically. I think what is hard is when people have to prioritize relationships, and if you’re coming in, and I’ve been part of couple situations where a third person has been brought in, but people are human, emotions exist. If people are experiencing jealousy or there’s only so much time to go around, oftentimes that third person doesn’t get prioritized or gets shortchanged or kind of is the collateral and the attempt to save the relationship. Or people aren’t on the same page in terms of expectations and that can be tough…It felt like the other person kind of was like, well my responsibly is to this person. And it’s like, right, but if they’re acting in ways that are fucked up, maybe address that? Whatever. And so that I guess there’s the risk of ganging up.

They conclude, however, “But I don’t know all sex is risky and all intimacy is emotionally risky. So might as well have fun with it?”

These interviewees spoke to both their initial interest and willingness to engage in sex and/or relationships with couples and the harm and disillusionment they experienced in how they were treated as the external member to the couple: the one without power. In some cases, the dynamic of the couple proved more difficult to navigate than felt worth the extra labor. In other cases, the bond of the couple created strengthened the experience of discomfort for those attempting engage with them. The couple’s bond and connection either felt too difficult to penetrate or, according to interviewees, the bond felt too precarious, for a specific member of couple, to permit third-person penetration. In the latter case, the relationship health of the couple and their respect for the third person became important factors. If couples were more concerned about their internal wants and needs than those of the third person, if they were not securely attached, or if they were working through their own relationship trauma (as suggested by a few interviewees), the third person entering these symbiosexual dynamics was at risk of greater harm and abuse than may be possible in one-to-one dynamics.

In sum, interviewee descriptions revealed a range of experiences specific to the dynamics of sex and relationships with couples. Both gratifying and undesirable experiences were described as more complex and intense than those offered in one-to-one dynamics. The broader spectrum of pleasure was weighed against a broader spectrum of harm. For many the benefits offered through symbiosexual dynamics outweighed the risks; for others, they did not.

## Discussion

### People Who Experience Symbiosexual Attraction Engage in Non-Monogamous, Multi-Person Sex and Relationship Dynamics and Those Specifically Attracted to Couples Engage in Sex and Relationships with Couples

I found that people with symbiosexual attraction engage in non-monogamous/multi-person dynamics. The percentage of people who reported engaging in these sex and relationship dynamics was surprisingly large considering that these dynamics are less normative. The primary recruitment efforts in *The Pleasure Study* toward non-monogamous populations likely skewed these results. More research is needed on symbiosexual behaviors to understand how their sexual and romantic choices are distributed in the general population. Since many people who experience attraction to couples reported engaging in threesomes, orgies, relationships with couples, three-way relationships, and open relationships, it is very possible that these experiences validated and informed their awareness of symbiosexual attraction. In Johnston’s (2024a) study on symbiosexual attraction, participants reported that feeling symbiosexual attraction was initially an unexpected or surprising experience. More research is needed on the evolution of awareness of symbiosexuality, and of non-normative sexual attractions and experiences in general, as well as how people make sense (or not) of such experiences.

While I found high rates of engagement of multi-person, non-monogamous dynamics in this population, the prevalence of these behaviors aligned closely with their level of social acceptability and ease of access. (For example, threesomes were more prevalent than orgies or three-way relationships.) Further, there was a notable gap between symbiosexual desire and sexual and romantic choices that would meet that desire. This gap may either speak to a less significant experience of desire for respondents than their single-object, gender-based attractions like gay or lesbian desires or this gap may speak to the structural difficulties of realizing multi-person, multi-directional attractions in our mononormative culture. The power of our culturally determined “charmed circle” (Rubin, 1984) of sexual and behavioral practices and the power of associated assumptions of monosexuality and monogamy may be greater than the power of symbiosexual desire.

I also found that people who experience attraction to couples specifically engage in sexual and romantic experiences with couples. While this seems intuitive, people report varied levels of engagement in these dynamics, some with frequency, some with little frequency. It was unsurprising that more people reported sexual experiences with couples than romantic experiences with couples as sexual experiences with couples are both easier to facilitate and more normalized in our “threesome” obsessed culture (Scoats, 2020). It was somewhat surprising, however, that of those who reported feeling attraction specifically to couples roughly 30% reported never having engaged in sexual experiences with couples and roughly 40% reported never having engaged in romantic experiences with couples. This disconnect between attraction and behavior may be due, in part, to lack of opportunity for these less normative experiences. This discrepancy may also be a result of the cultural stigma, both broadly and particularly within the polyamorous community, associated with engaging in sex and relationships with established couples (Johnston, 2024c). Further, cultural reverence for and protection of monogamous couples and the stigmas associated with those who “disrupt” this bond (e.g., use of the term “homewrecker”) (Hardy & Easton, 2017; Willey, 2016) may be deterring those with a desire to engage with couples from acting on these desires or even fully acknowledging them. More research is needed on why some people who experience symbiosexual attraction do not engage in sex and relationships with couples and how they make sense of this unrealized attraction.

### People Report Gratifying and Undesirable Experiences in Sex and Relationships with Couples

I found that people report both gratifying and undesirable experiences in sex and relationships with couples. The gratifying experiences described, including heightened pleasure and fun, the opportunity for playing with roles, gender expansiveness, the opportunity to be objectified or have the “unicorn” experience, heightened intimacy and closeness, the opportunity for healing/transformation, and even associated challenges described as part of an overall gratifying experience, suggest that the blanket stigma within the polyamorous community that dynamics with couples are harmful and unethical may be inaccurate or overemphasized.

Interviewees specifically described gratifying experiences that offered stronger and more varied power dynamics. This appeal may speak to an orientation toward power itself (Sprott & Williams, 2019) which is better accessed through symbiosexual dynamics. Symbiosexual desire may be oriented toward the power that is unevenly distributed in favor of the couple or toward subversion of the power of the couple in dynamics where the third person may actually hold more power in the “three-way marketplace” (Beggan, 2020).

Further, gender play in symbiosexual dynamics offered potential for pleasure and satisfaction in the subversion of power. As Schippers (2016) argues, the threesome imaginary, as a reflection of “gendered power relations,” has the potential to disrupt and undo existing power relations between bodies (p. 143). Gender play and undoing of gender in symbiosexual dynamics may grant people access to pleasure that has been systematically denied to them through the doing of gender (West & Zimmerman, 1987) and the experience of being gendered in both two-person and three-person sex and relationships which operate to reaffirm hegemonic masculinity (Schippers, 2016; Ward, 2020).

Whether relevant to power play or not, interviewees also described pleasure in choosing an experience of objectification in symbiosexual dynamics. The distinction between being objectified and choosing objectification lacks attention in polyamorous communities where there is an assumption of disempowered, harmful objectification for a third person joining a couple (Johnston, 2024c).

Further, findings of heightened pleasure, opportunity for authenticity (including authenticity of gender expression and sexuality), and personal transcendence and healing in symbiosexual dynamics may have important therapeutic implications and may particularly hold relevance to the psychological concept of triangulation. While it is widely accepted that people develop attachment representations though their relationships with their primary caregivers, some family therapy scholars argue that people develop attachment representations not only through their individual attachments with their caregivers but through the relationship *between* their caregivers (Dallos & Vetete, 2012). It is within this system of three, the individual and the dyad, that adults who experience symbiosexual attraction may seek to re-engage with the healthy, loving attachments that nurtured them as a child. Conversely, sexual and romantic dynamics with couples may offer a first container for experiencing the necessary nurturance and stabilization that builds self-concept and relational resilience, as well as fosters healthy attachment patterns. Recent discourse on the relationship between non-monogamy, attachment, and trauma supports these possibilities (Fern, 2020); however, more investigation is needed. Further, research specifically on the relationship between triangulation, attachment, and symbiosexuality is needed.

Interviewees reported gratifying experiences with couples despite structural challenges. In some cases, the work of “undoing” did not override pleasure. Willey (2016) talks about this undoing as an uphill battle against compulsory monogamy, our assumptions of exclusivity and twoness as “natural” to human sexuality, bonding, and belonging. Those who experience symbiosexual attraction and seek and/or engage in gratifying experiences with couples should be recognized, validated, and supported both within the polyamorous community and within our broader Westernized world.

More research is needed on the unique and heightened positive outcomes associated with symbiosexual dynamics, particularly for the themes that emerged in this study around heightened pleasure, opportunity for authenticity (including authenticity of gender expression and sexuality), and personal transcendence and healing. These experiences have implications for pathways to sexual pleasure and fulfillment that remain under investigated in current discourses of desire and pleasure—discourses that center gender-based sexualities and desires and behaviors that are specifically “sexual.” There are more complex, dimensional possibilities in what may be labeled as “erotic” (Willey, 2016); symbiosexuality is one such possibility that needs more investigation as a pathway to pleasure and fulfillment.

On the other hand, the undesirable experiences described by interviewees, including difficult navigation, upset/uncomfortable feelings, couple’s hierarchy, and dehumanization of the third person, were consistent with the reputation and stigma of unicorn dynamics in the polyamorous community (Johnston, 2024c). Feelings of discomfort in these dynamics may not only apply to what is happening between the couple and the third person but how what is happening aligns with or challenges accepted gender roles within the dynamics of group sex. This finding is supported by recent studies on how attitudes toward threesomes differ based on gender and align with gendered sexual scripts that privilege FFM (female, female, male) threesomes above other configurations (Thompson & Byers, 2017, 2021; Thompson et al., 2021). Evidence of disrespect and emotional harm in these descriptions warrant further investigation.

Whether they seek support and information in the polyamory communities or not, those who experience symbiosexual desire and want to engage in sex and relationships with couples need effective tools and support to facilitate such interactions should they so choose. Again, considering the concept of triangulation, if these experiences can offer a container for something uniquely beneficial, they can also foster experiences that are uniquely harmful. While beyond the scope of this study, many of the participants who engaged in sex and relationships with couples included tip, tricks, and advice they had learned for engaging in these dynamics to prevent harm and abuse. Future research should examine effective strategies for safe, healthy engagement in these dynamics.

## Limitations

There were several limitations of this exploratory study of symbiosexual experiences and symbiosexual informed sexualities. I analyzed qualitative and quantitative data collected from *The Pleasure Study* (Harvey et al., 2023), which used convenience and snowball sampling. Therefore, the sample was biased by those who were specifically recruited by *The Pleasure Study* (queer and non-monogamous populations), those who self-selected to participate in a survey and an interview about sex and sexuality, and those who chose not to answer specific questions. Any conclusions drawn from this data are tentative and preliminary, and at risk of incorrect assessment due to the biases mentioned above.

## Conclusion

This study provided account and recognition for the complex lived experiences of sexuality for those who experience symbiosexual attraction. Participants reported a variety of multi-person sexual and romantic experiences, the most fascinating of which were those of the unique and/or heightened positive outcomes made possible through experiences with couples. These accounts challenge dominant dyadic and triadic sexual scripts (Gagnon & Simon, 1973) that center the roles of individual bodies and highlight the erotic possibilities found in the interplay of actors who are in relationships. Comparing survey responses and these qualitative accounts undoes the implicit and explicit links between sexuality and danger typical to investigations of “non-normative” multi-person sexual behaviors (Rubin, 1984; Saperstein & Westbrook, 2021; Westbrook & Saperstein, 2015) and fortifies and validates links between multi-person, multi-directional sexualities, and pleasure.

This work also challenges assumptions of monosexism and single-object desires and assumptions of an inherent danger in validating such desires both within our mononormative culture and within the non-monogamous communities that should be a refuge for symbiosexualities. As Angel reminds us “all sex is risky…So might as well have fun with it.” For this study population, fun as well as the most pleasure and satisfaction was found outside of gender-based, subject-object sexual attractions and experiences. It was in sex and relationships with people in relationship that some participants were able to connect more fully with their humanity, sexuality, and pleasure.

## Survey Questions Analyzed

**Table:**

**Survey Questions** Gender Identity Label I most commonly use (select all that apply)—Selected Choice
• The sexual orientation label that best describes me is (select all that apply)—Selected Choice • Do you identify as monogamous, polyamorous, other (please specify)
• Have you ever felt sexually/romantically attracted to a couple (two people and their relationship together, not each of them individually)?
Regarding your attraction to couples (two people in a relationship together)…
I have had sexual experiences with couples...(never, once, a few times, sometimes, often) I have had romantic experiences with couples...(never, once, a few times, sometimes, often)

*Note.* Questions only include a small portion of the survey questions from Stage 2 of The Pleasure Study (Harvey et al., 2023).

## Interview Questions

Interview Guide

Section 2: Attractions

People attracted to couples

- Describe your experiences with attraction to couples*
- sexual and dating experiences?
- Feelings about those experiences, positive, negative? Why?
- Ask them to elaborate on any difficulties with couples

*If they use the term “**unicorn**,” ask them to elaborate on what that term means to them?

*Note.* Questions only include a small portion of the interview questions from Sects. 2 of the interview guide from Stage 2 of The Pleasure Study (Harvey et al., 2023).

## Footnotes

1 Compersion is a term for the range of positive emotions such as joy, arousal, and contentment that some people experience as a witness to intimate relations between others, with whom they also share intimate connections (Thouin & Flicker, 2023).
